# Validating a widely used measure of frailty: are all sub-components necessary? Evidence from the Whitehall II cohort study

**DOI:** 10.1007/s11357-012-9446-2

**Published:** 2012-07-08

**Authors:** Kim Bouillon, Severine Sabia, Markus Jokela, Catharine R. Gale, Archana Singh-Manoux, Martin J. Shipley, Mika Kivimäki, G. David Batty

**Affiliations:** 1 Department of Epidemiology and Public Health, University College London, 1-19 Torrington Place, London, WC1E 6BT UK; 2 Institute of Behavioural Sciences, University of Helsinki, Helsinki, Finland; 3 MRC Lifecourse Epidemiology Unit, University of Southampton, Southampton, UK; 4 Centre for Cognitive Ageing and Cognitive Epidemiology, University of Edinburgh, Edinburgh, UK; 5 INSERM U1018, Centre for Research in Epidemiology & Population Health, Villejuif, France; 6 Centre de Gérontologie, Hôpital Ste Périne, AP-HP, Paris, France; 7 Finnish Institute of Occupational Health, Helsinki, Finland

**Keywords:** Frail elderly, Frailty, Validation, Prediction, Cohort study, Hospitalization

## Abstract

There is growing interest in the measurement of frailty in older age. The most widely used measure (Fried) characterizes this syndrome using five components: exhaustion, physical activity, walking speed, grip strength, and weight loss. These components overlap, raising the possibility of using fewer, and therefore making the device more time- and cost-efficient. The analytic sample was 5,169 individuals (1,419 women) from the British Whitehall II cohort study, aged 55 to 79 years in 2007–2009. Hospitalization data were accessed through English national records (mean follow-up 15.2 months). Age- and sex-adjusted Cox models showed that all components were significantly associated with hospitalization, the hazard ratios (HR) ranging from 1.18 (95 % confidence interval = 0.98, 1.41) for grip strength to 1.60 (1.35, 1.90) for usual walking speed. Some attenuation of these effects was apparent following mutual adjustment for frailty components, but the rank order of the strength of association remained unchanged. We observed a dose–response relationship between the number of frailty components and the risk for hospitalization [1 component—HR = 1.10 (0.96, 1.26); 2—HR = 1.52 (1.26, 1.83); 3–5—HR = 2.41 (1.84, 3.16), *P* trend <0.0001]. A concordance index used to evaluate the predictive power for hospital admissions of individual components and the full scale was modest in magnitude (range 0.57 to 0.58). Our results support the validity of the multi-component frailty measure, but the predictive performance of the measure is poor.

**Electronic supplementary material:**

The online version of this article (doi:10.1007/s11357-012-9446-2) contains supplementary material, which is available to authorized users.

## Introduction

Frailty is a clinically recognized geriatric syndrome characterized by age-related declines in functional reserves across an array of physiologic systems (Fried et al. 2001). In older adults, it is associated with multiple adverse health outcomes such as falls, fracture, disability, hospitalization, and mortality (Cawthon et al. 2007; Fried et al. 2001). There is evidence that frailty may be prevented (Boyd et al. 2009; Tan et al. 2009) and perhaps even reversed with appropriate intervention (Faber et al. 2006; Kenny et al. 2010; Peterson et al. 2007; Srinivas-Shankar et al. 2010).

Recent systematic literature reviews identified more than 20 frailty measures (de Vries et al. 2011; Sternberg et al. 2011), among which that developed by Fried and colleagues (Fried et al. 2001) is the most widely utilized. Comprising five components—weight loss, exhaustion, low physical activity, slow walking speed at usual pace, and low grip strength—this scale has been validated against subsequent health outcomes in a series of studies drawn from a range of diverse populations (Al Snih et al. 2009; Avila-Funes et al. 2008; Bandeen-Roche et al. 2006; Cawthon et al. 2007; Ensrud et al. 2007; Fairhall et al. 2011; Kiely et al. 2009; Kulminski et al. 2008; Rochat et al. 2010; Romero-Ortuno et al. 2010; Seematter-Bagnoud et al. 2010; Wong et al. 2010; Woods et al. 2005).

While it is assumed that the measurement of frailty needs to include multiple components, these inevitably overlap. Using fewer components would be more time- and cost-efficient. Although studies using the Fried frailty scale have generally shown that the greater the number of frailty components used the higher the risk of a given adverse health outcome (Avila-Funes et al. 2008; Bandeen-Roche et al. 2006; Cawthon et al. 2007; Ensrud et al. 2008; Fried et al. 2001; Kulminski et al. 2008), it remains unclear whether all components of the scale contribute to associations with health outcomes or whether some of them are redundant. Accordingly, for the first time to our knowledge, we compared the prediction accuracy of multi-component measures of frailty for total hospitalizations with a single-component measure.

## Materials and methods

### Study sample

The Whitehall II study is an ongoing, cohort study in which 10,308 (67 % men) London-based British civil servants aged 35–55 years were recruited in 1985 (Marmot and Brunner 2005). The first examination (phase 1), which took place during 1985–1988, comprised a clinical examination and self-administered questionnaire. Subsequent phases of data collection have alternated between postal questionnaire alone [phases 2 (1988–1990), 4 (1995–1996), 6 (2001) and 8 (2006)], and postal questionnaire accompanied by a clinical examination [phases 3 (1991–1993), 5 (1997–1999), 7 (2002–2004) and 9 (2007–2009)].

**Fig. 1:**
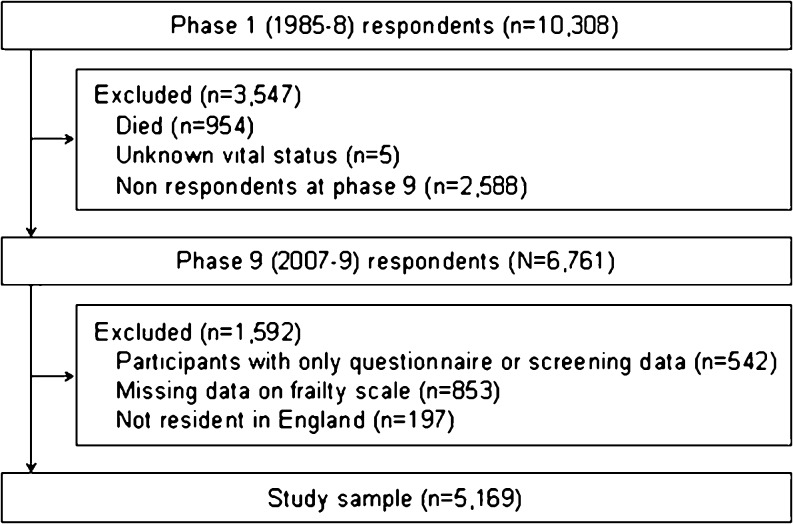
Flow of study participants through the Whitehall II study, UK, 1989–2010

For the current analyses, we used data drawn from phase 9 when frailty was first measured; this therefore represents our “baseline” for the present analyses. Of 10,308 study members at recruitment, 6,761 participated at phase 9, 2,588 were non-responders, 954 had died and the vital status of five was unknown. Of the 6,761 participants at phase 9, complete data for the frailty components and hospitalizations were available for 5,169 (74 % men). This constituted the study sample. The flow of participants through the study is depicted in Fig. 1.

Ethical approval for the Whitehall II study was obtained from the University College London Medical School Committee on the ethics of human research.

### Operationalization of frailty measure

The Fried frailty measure comprises the following components (Fried et al. 2001):

1. *Exhaustion*: defined using two items drawn from the Center for Epidemiology Studies—Depression (CES-D) scale (Radloff 1977): “I felt that everything I did was an effort in the last week” and “I could not get going in the last week”. If participants answered “occasionally or moderate amount of the time (3–4 days)” or “most or all of the time (5–7 days)” to either of these items, they were categorized as exhausted. If they answered “rarely or none of the time (<1 day)” or “some or a little of the time (1–2 days)”, they were categorized as not exhausted.
2. *Physical activity*: based on a modified version of the Minnesota leisure-time physical activity questionnaire (Folsom et al. 1985; Singh-Manoux et al. 2005) which ascertains the frequency and duration of participation in 20 different activities (e.g., running, cycling, other sports, housework, and gardening activities). Total hours per week were calculated for each activity and a metabolic equivalent (MET) value was assigned to each based on an existing compendium (Ainsworth et al. 1993). Energy expenditure (kcal/week) was calculated for each participant; low levels of physical activity were denoted by an expenditure of <383 kcal/week (men) and 270 (women) (Fried et al. 2001).
3. *Walking speed*: based on the duration of walking a distance of 8-ft (2.4 m) at usual pace. Established cut-offs are based on results for a 15-ft (4.6 m) walking test (Fried et al. 2001). Accordingly, following recomputation, participants were categorized as having slow walking speed when time to walk 8-ft was ≥3.73 s (for men with height ≤173 cm or women with height ≤159 cm) or ≥3.20 s (for men with height >173 cm or women with height >159 cm).
4. *Grip strength*: measured in kilograms using the Smedley hand grip dynamometer. Categorizations were stratified by gender and body mass index (BMI) (Fried et al. 2001). For men, low grip strength was ≤29 kg (BMI ≤24 kg/m^2^), ≤30 (BMI 24.1–28), and ≤32 (BMI > 28). For women, low grip strength was ≤17 (BMI ≤ 23), ≤17.3 (BMI 23.1–26), ≤18 (BMI 26.1–29), and ≤21 (BMI > 29).
5. *Weight loss*: Prior definitions of weight loss in the context of frailty have defined it as being unintentional and 5 % or more over the previous year (Fried et al. 2001). We did not have weight loss in the previous year, so we instead utilized a cut-off of 10 % in accordance with that in the Women’s Health Aging Study-I (Boyd et al. 2005).

A total frailty score was calculated by allocating a value of 1 to each of the above criteria, resulting in a range of 0 to 5. Participants were classified as *frail* if they had at least three out of five of the frailty components, as *pre-frail* if they had 1–2, and as *non-frail* if they had none of these components.

### Outcome

Information on the first hospitalization was prospectively ascertained from phase 9 (October 10, 2007) to January 31, 2010 by linkage of study members to the Hospital Episode Statistics (HES), a data registry including information on all admissions to National Health Service hospitals in England (The NHS Information Centre for health and social care 2011).

### Statistical analysis

Incidence curves for hospitalization according to frailty status were produced using Kaplan–Meier plots (Kaplan and Meier 1958) and the log-rank test (Peto and Peto 1972). Having first ascertained that the proportional hazards assumptions had not been violated, hazard ratios (HR) and accompanying 95 % confidence intervals (CI) for the associations of frailty (and its individual components) with all hospitalizations combined were computed using Cox proportional hazard regression models (Cox 1972). Given that there was no evidence that the relation between frailty and hospitalization was modified by gender or age (all *P* values for interaction >0.45), data were pooled and adjusted for age and gender.

We first examined whether individual frailty markers were associated with the risk of hospitalization. Second, in order to explore whether a single component was responsible for generating the association between the overall frailty scale and the risk of hospitalization, we examined the cumulative effect of frailty markers in the prediction of hospitalization by creating a frailty score ranging from 0 (no frailty) to 5. We then studied the effect of number and combinations of frailty components on the risk of hospitalization in two separate models. We also conducted a subgroup analysis among study participants who were negative for a given frailty component to estimate cumulative effects (0 to 4) of other frailty components in the prediction of adverse health outcomes. In all analyses, the reference group was that with no apparent frailty.

To evaluate the predictive power for each individual component and the full frailty scale, we calculated Harrell’s *C* concordance statistic (Harrell, Jr. et al. 1996), which is equivalent to the area under the curve statistic for receiver-operating characteristic plots but allows the calculation of concordance in each survival model. It estimates the concordance between the predicted failure order of a pair of subjects and the observed order. We split the analytic sample into “derivation” and “validation” datasets of equal size after stratification by age and sex. We then fitted age- and sex-adjusted models in the derivation dataset and evaluated the performance of the models in the validation dataset (Newson 2010).

Descriptive analyses and Cox proportional hazards models were performed using SAS version 9.1. Calculations of Harrell’s *C* concordance statistic were performed using Stata version 10.0.

## Results

### Study participants and missing data

Compared with participants alive at phase 9 but excluded (owing to unknown vital status, non-participation, missing data on the frailty scale, and living outside of England) (*n* = 4,153), people in the analytic sample (*n* = 5,169) were on average 0.7 years younger, less likely to be female (27.5 % versus 39.7 %), and of low socioeconomic status (3.9 % versus 12.4 %).

In Table 1, we report the baseline characteristics of study members according to hospitalization. Of the 5,169 participants, 22.3 % had at least one hospitalization episode during the follow-up (range 0.03 to 28.13 months; mean = 15.17). In comparison with non-hospitalized participants, hospitalized participants were more likely to be older, positive for each frailty component, and classified as frail or pre-frail.

**Table 1:** Baseline characteristics of the 5,169 study participants according to hospitalization during follow-up, Whitehall II study, UK, 2007–2010

	Hospitalization		*P* value^a^
	*n* (%)		
	No	Yes	
*N*	4,019	1,150	
Age in years [mean (SD)]	65.4 (5.8)	67.2 (6.0)	<0.0001
Women	1,104 (27.5)	315 (27.4)	0.96
Frailty components			
Exhaustion	402 (10.0)	152 (13.2)	0.002
Low physical activity	875 (21.8)	301 (26.2)	0.002
Slow walking speed	340 (8.5)	163 (14.2)	<0.0001
Low grip strength	373 (9.3)	139 (12.1)	0.005
Weight loss	135 (3.4)	54 (4.7)	0.03
Frailty status			<0.0001
Non-frail	2,415 (60.1)	614 (53.4)	
Pre-frail	1,517 (37.8)	476 (41.4)	
Frail	87 (2.1)	60 (5.2)	

^a^ *P* value for heterogeneity except for frailty status where *P* value is for trend

### Association between frailty and future risk of hospitalization

**Fig. 2:**
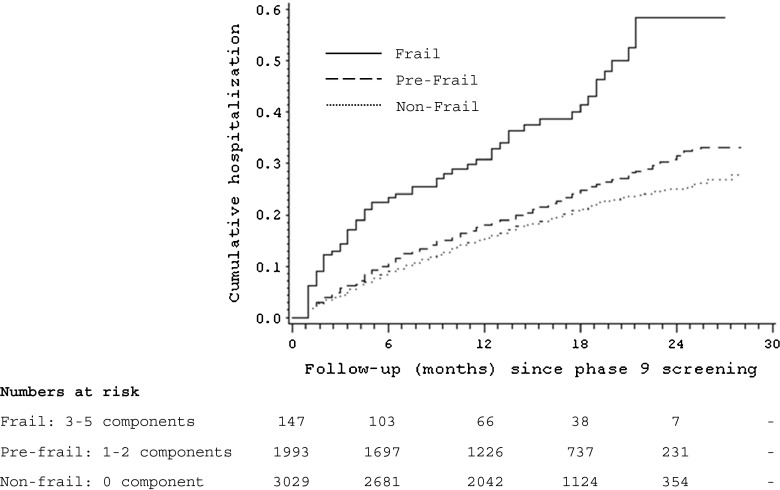
Kaplan–Meier curves showing the probability of future hospitalization by frailty status, Whitehall II study, UK, 2007–2010

Kaplan–Meier curves (Fig. 2) show that the cumulative hospitalization rate was higher among the frail group compared with their non-frail counterparts (*P* value for difference <0.0001). In age- and sex-adjusted analyses, with the non-frail group as the referent, the frail group had an elevated hazard ratio for hospitalization of 2.40 [95 % confidence interval (CI) = 1.83, 3.14] while for the pre-frail group it was 1.20 (95 % CI = 1.06, 1.35).

### Association between single components of frailty and future risk of hospitalization

Table 2 illustrates the results of the association between individual frailty components and the risk of hospitalization. All five components were significantly associated with hospitalization, with the age- and sex-adjusted hazard ratios ranging from 1.18 (95 % CI = 0.98, 1.41) for grip strength to 1.60 (95 % CI = 1.35, 1.90) for walking speed. Some attenuation was apparent following adjustment for other components, but the rank order of the strength of association remained unchanged.

**Table 2:** Hazard ratios (95 % confidence interval) for the association of individual frailty components with hospitalization (*n* = 5,169), Whitehall II study, UK, 2007–2010

	Hospitalization		HR [95 % CI]	HR [95 % CI]
	*N* (%)		Sex- and age-adjusted	Fully adjusted^a^
	No	Yes		
Exhaustion				
No	3,617 (90.0)	998 (86.8)	1 [Ref]	1
Yes	402 (10.0)	152 (13.2)	1.38 [1.17, 1.64]	1.30 [1.10, 1.55]
Low physical activity				
No	3,144 (78.2)	849 (73.8)	1	1
Yes	875 (21.8)	301 (26.2)	1.26 [1.10, 1.44]	1.19 [1.04, 1.36]
Slow walking speed				
No	3,679 (91.5)	987 (85.8)	1	1
Yes	340 (8.5)	163 (14.2)	1.60 [1.35, 1.90]	1.52 [1.28, 1.80]
Low grip strength				
No	3,646 (90.7)	1,011 (87.9)	1	1
Yes	373 (9.3)	139 (12.1)	1.18 [0.98, 1.41]	1.07 [0.89, 1.28]
Weight loss				
No	3,884 (96.6)	1,096 (95.3)	1	1
Yes	135 (3.4)	54 (4.7)	1.41 [1.07, 1.86]	1.34 [1.02, 1.77]

^a^Adjustment for sex, age, exhaustion, physical activity, walking speed, grip strength, and weight loss

### Cumulative effect of frailty markers and the risk of hospitalization

**Fig. 3:**
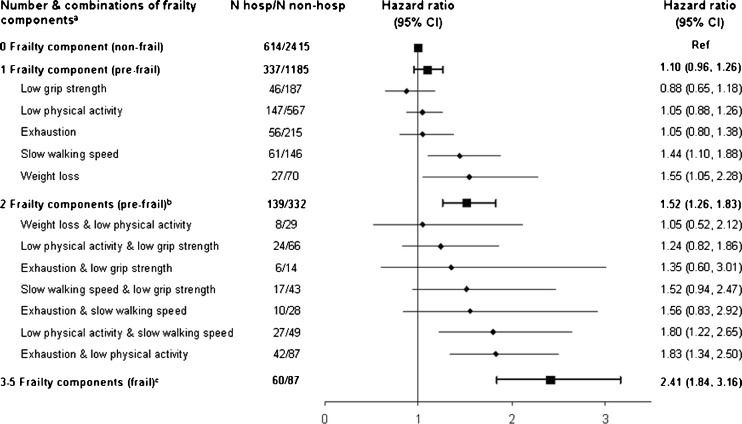
Hazard ratios (95 % confidence interval) for the association of combinations of frailty components with later hospitalization, Whitehall II study, UK, 2007–2010. ^a^Results from two models: one with different combinations included in the model (*diamonds*); the other one with number of frailty components included in the model (*squares*). All analyses were adjusted for age at baseline and sex. The reference group was those with no frailty component. ^b^Three combinations were not reported owing to too few hospitalizations: weight loss and slow walking speed (*n* = 0), weight loss and exhaustion (*n* = 2), and weight loss and low grip strength (*n* = 3). ^c^Owing to low numbers, participants with three to five frailty components were collapsed

Figure 3 shows a dose–response relationship between the risk of hospitalization and the number frailty components: the hazard ratios for hospitalization ranged from 1.10 (95 % CI = 0.96, 1.26) (any single frailty component) to 2.41 (95 % CI = 1.84, 3.16) (3–5 frailty components). Figure 3 also displays hazard ratios and their 95 % CIs for hospitalization according to different combinations of indicators included in the frailty scale when the scores were less than 3. Among study members with one frailty component only, the strength and the rank of association of each separate frailty component was slightly different from those reported in Table 2 where this estimation was carried out among the study participants with a frailty score of one or more. When we examined the possible combinations of two items from the frailty scale, there were very few study members with weight loss; therefore, three combinations were not represented. Two (low physical activity and slow walking speed; exhaustion and low physical activity) of a possible 10 combinations of those with two frailty indicators had very similar and strong associations (hazard ratios ranging from 1.80 to 1.83) with hospitalization.

In Table 3, we present the results of the association between the number of frailty components with the risk for hospitalization stratified by the presence of individual frailty components. Within each stratum, we still observed dose–response associations between the frailty score and the risk for hospitalization.

**Table 3:** Hazard ratios (95 % confidence interval) for the association of number of frailty components with hospitalization, stratified by individual components, Whitehall II study, UK, 2007–2010

	*N* hosp	*N* non-hosp	HR [95 % CI]		*N* hosp	*N* non-hosp	HR [95 % CI]
Exhaustion = no	998	3,617		Exhaustion = yes	152	402	
0	614	2,415	1 [Ref]	0	56	215	1 [Ref]
1	281	970	1.11 [0.96, 1.28]	1	60	136	1.62 [1.12, 2.33]
2	79	196	1.40 [1.10, 1.77]	2	24	39	2.30 [1.41, 3.74]
3–4	24	36	2.09 [1.39, 3.16]	3–4	12	12	3.72 [1.97, 7.01]
*P* value for trend			0.0001	*P* value for trend			<0.0001
Low physical activity = no	849	3,144		Low physical activity = yes	301	875	
0	614	2,415	1 [Ref]	0	147	567	1 [Ref]
1	190	618	1.14 [0.96, 1.34]	1	101	231	1.52 [1.17, 1.96]
2	38	101	1.40 [1.01, 1.96]	2	41	64	2.25 [1.58, 3.21]
3–4	7	10	2.22 [1.05, 4.69]	3–4	12	13	3.61 [2.00, 6.55]
*P* value for trend			0.005	*P* value for trend			<0.0001
Slow walking speed = no	987	3,679		Slow walking speed = yes	163	340	
0	614	2,415	1 [Ref]	0	61	146	1 [Ref]
1	276	1,039	1.05 [0.91, 1.21]	1	54	124	1.13 [0.78, 1.63]
2	85	208	1.47 [1.17, 1.85]	2	36	57	1.52 [1.00, 2.30]
3–4	12	17	2.58 [1.46, 4.57]	3–4	12	13	2.46 [1.32, 4.58]
*P* value for trend			0.0004	*P* value for trend			0.004
Low grip strength = no	1,011	3,646		Low grip strength = yes	139	373	
0	614	2,415	1 [Ref]	0	46	187	1 [Ref]
1	291	998	1.15 [1.00, 1.32]	1	50	128	1.58 [1.06, 2.36]
2	89	204	1.61 [1.29, 2.02]	2	33	49	2.56 [1.63, 4.01]
3–4	17	29	2.48 [1.52, 4.03]	3–4	10	9	4.93 [2.47, 9.84]
*P* value for trend			<0.0001	*P* value for trend			<0.0001
Weight loss = no	1,096	3,884		Weight loss = yes	54	135	
0	614	2,415	1 [Ref]	0	27	70	1 [Ref]
1	310	1,115	1.08 [0.94, 1.23]	1	13	45	0.69 [0.35, 1.35]
2	126	287	1.59 [1.31, 1.92]	2	10	13	2.09 [0.99, 4.39]
3–4	46	67	2.33 [1.72, 3.16]	3–4	4	7	1.54 [0.53, 4.49]
*P* value for trend			<0.0001	*P* value for trend			0.19

### Predictive power of single- and multi-component measures for hospitalization

Harrell’s *C* concordance statistic for individual frailty components and the full frailty scale varied very little: 0.57 (95 % CI = 0.55, 0.60) for grip strength and 0.58 (95 % CI = 0.56, 0.61) for exhaustion and the full frailty scale. The difference of Harrell’s concordance indices between pairs of individual components and the full scale was not statistically significant at conventional levels (*P* values >0.06; see Online appendix).

## Discussion

The main objective of this study was to examine whether the five components included in the Fried frailty scale were equally related to the risk of hospitalization or whether one single component, or a combination, had the same utility as the full scale.

Although the dose–response relationship between the number of frailty components and the risk of adverse health outcomes (Avila-Funes et al. 2008; Bandeen-Roche et al. 2006; Cawthon et al. 2007; Ensrud et al. 2008; Fried et al. 2001; Kulminski et al. 2008) is well known, our results add some novel findings to this literature. First, we show that all five frailty components—exhaustion, low physical activity, slow walking speed, low grip strength, and weight loss—are independently associated with hospitalization with none of them being redundant. Thus, these analyses support the hypothesis that several components are required to measure frailty (Fried et al. 2001; Rockwood 2005). Our results are consistent with those from a previous study (Rothman et al. 2008) where the authors found that slow walking speed was the strongest, and low grip strength the weakest, predictors of hospitalization.

Second, we formally tested the predictive performance of the individual frailty components compared with the full frailty scale. Harrell’s *C* concordance statistic varied between 0.57 and 0.58 (0.50 indicates that the prediction does not differ from chance), suggesting that neither the components nor the full scale were adequate prediction tools for hospitalization in the clinical settings. This probably indicates that frailty and its components capture only a limited range of the conditions leading to hospitalization. Third, the absence of difference in predictive performance between individual components and the full scale suggest that measuring only one component of frailty enables an equally precise prediction of hospitalization as the full scale; other analyses conducted in this study did not support this conclusion. Importantly, we found that within the group of individuals with a frailty component those who additionally had other components were up to 4.9 times more likely to experience hospitalization at follow-up compared with those with no additional frailty components. Thus, the frailty measure seemed to stratify risk even within the group of individuals with an individual frailty component.

The main strength of our study resides in using an objective and national database (British National Health Service) to derive our outcome. Therefore, it is unlikely to be subject to reporting bias. A limitation, shared with many studies in this field of research, is a departure from the original frailty scale. This was particularly the case with weight loss because weight in the previous year was not available in our study. As many studies on frailty, including ours, are analyses of existing cohorts primarily set up for other purposes, assessment of frailty components tends to differ between them. Nonetheless, effort should be made to use a standardized definition in order to allow direct comparisons of results between different populations. Furthermore, because our study sample consisted predominantly of white collar workers aged from 55 to 79 years (mean age = 65.8), this may limit the generalizability of our findings.

In conclusion, our results indicate that a composite measure of frailty proposed by Fried is related to future risk of hospitalization but shows poor performance as a predictive tool. Much previous work in this domain is based on elderly individuals. That the frailty scale and its individual components are prospectively associated with hospitalization in our cohort, where participants were aged 55–79 years at baseline, suggests that the scale could be used to reliably monitor frailty status of adults in middle and early old age.

## Electronic supplementary material

Below is the link to the electronic supplementary material.

ESM 1 (DOC 50 kb)
