# Supplementary material for: Validating a widely used measure of frailty: are all sub-components necessary? Evidence from the Whitehall II cohort study
Source: Age (Dordr). 2012 Jul 8;35(4):1457–65. doi: 10.1007/s11357-012-9446-2 (PMC3705104; doi:10.1007/s11357-012-9446-2)
Supplement: Supplementary file 1 — (DOC 50 kb) [file 11357_2012_9446_MOESM1_ESM.doc]

Supplementary material

Table. Performance of models in the prediction for hospitalization including individual components and the Fried frailty scale, Whitehall II study, UK, 2007-2010

| Model | Harrell’s c statistic [95% CI] | P-value* |
| --- | --- | --- |
| M1: Age, sex | 0.574 [0.549, 0.600] | 0.196 |
| M2: M1 + exhaustion (yes/no) | 0.583 [0.558, 0.607] | 0.401 |
| M3: M1 + low physical activity (yes/no) | 0.579 [0.554, 0.605] | 0.903 |
| M4: M1 + slow walking speed (yes/no) | 0.578 [0.553, 0.604] | 0.841 |
| M5: M1+ low grip strength (yes/no) | 0.573 [0.548, 0.598] | 0.056 |
| M6: M1 + weight loss (yes/no) | 0.575 [0.549, 0.600] | 0.236 |
| M7: M1 + Fried frailty scale (3-5 versus 0-2) | 0.579 [0.554, 0.604] | - |

*P-value comparing the predictive values of M1 to M6 with M7.

**Article title**: Validating a Widely Used Measure of Frailty: Are All Sub-components Necessary? Evidence from the Whitehall II cohort study

**Journal name**: Age

**Author names**: Kim Bouillon, Severine Sabia, Markus Jokela, Catharine R Gale, Archana Singh-Manoux, Martin Shipley, Mika Kivimäki, G. David Batty.

**Affiliation and email address of the corresponding author**: Dr. Kim Bouillon, Department of Epidemiology and Public Health, University College London; E-mail: [kim.bouillon.09@ucl.ac.uk](mailto:kim.bouillon.09@ucl.ac.uk)
